# Alessandro Rosina, Sergio Sorgi: Il futuro che (non) c’è. Costruire un domani migliore con la demografia. The future that is(n’t). Building a better future with demography

**DOI:** 10.1186/s41118-018-0034-x

**Published:** 2018-05-10

**Authors:** Alessandra Minello

**Affiliations:** Universita degli Studi di Firenze, Florence, Italy

This book aims to show the relevance of demography when dealing with important spheres in the public debate: welfare, work, market, innovation, culture, democracy, and sustainable development. Its contribution is to approach each topic by considering the changing dynamics of the Italian population. The authors propose solutions to the present and future challenges of the country encouraging mechanisms of positive growth.

It is essential to take into account intergenerational changes and the changing life of single persons, according to the authors. For this reason, they present a clear picture of how each of their themes develops across population groups such as young people, women, immigrants, and seniors. The book stresses the importance of finding a way to deal with the growing uncertainty of modern life and of having the necessary instruments to make solid decisions about the future. Demography has a role in helping to deal with important issues such as the connection between longer life expectancy and the need to improve overall subjective and objective wellbeing.

The first theme of the book is welfare. Changes in the composition of the population (e.g., longer life expectancy and new forms of the family) must be taken into account to make a new generation of welfare available for all the population, not only to cover the needs of poor people. Citizens having a sense of responsibility, mechanisms to reward good practices, and the concept of networks appear determinant in the future development of welfare. Promoting lifelong wellbeing, understood as a mix of indicators, must be the aim of social policies. Measurability of the results obtained must be guaranteed. Encouraging participation and reinforcing economic security and the sense of social belonging should be, according to the authors, the other priorities of the future.

The second theme is work. Diverse competences, experiences, aims, and expectations to various phases of life should be emphasized by society. Young people should be encouraged and actively supported, guaranteeing chances of success and development of personal potential which should not be hindered by characteristics such as gender or social class. Those over 55 and both out of the labor market and out of the system of lifelong learning are also an issue. Their value should be increased, promoting individual capacities, investing in human capital, taking advantage of their experience, and considering them not as a whole, but with individual needs and potentials. Overall, the key word is “growth”: not in terms of quantity but in terms of quality, especially with reference to the need to improve the wellbeing of the young (and old) generations.

The third theme is the market. Age, gender, wages, types of job, and types of families are only some of the features to be considered to offer goods and services to citizens. As an example, changing male and female tastes and attitudes should drive a change in the market offer. NEETs (namely, young individuals not in employment, education, or training) and seniors are two vulnerable groups. Families are changing too, with an increasing number of single families. The silver economy now deserves a leading role in defining the market. Overall, the picture of market offer should take into account uncertainty about the future, distrust on institutions, and tendency to accumulate. At the same time, the present is characterized by shortages of resources, which lead to greater consciousness, new forms of sobriety, and new forms of sharing.

The fourth theme is innovation. Innovation is presented as important to deal with uncertainty by proposing plans, goals, and methods to measure whether they are achieved and, eventually, to correct them. Scarcity of resources has brought decisions far from citizens (and from the cities) back to central level. However, some local innovation must be considered and enhanced if it is best practice. Inclusion is fundamental: the young, women, seniors, and vulnerable people (with health or financial issues) must be included in the labor market and must participate in the creation of the future, with a specific orientation towards general wellbeing, at both the individual and the community levels.

The fifth theme is culture. It is a means of adapting to the environment and of modifying it to improve living conditions and prosperity by enhancing resources and spaces. Immigration and gender are central topics. Increasing life expectancy, changes in fertility, and a new role for women in society and the labor market have made change a characteristic of recent decades, far from any equilibrium. Women have modified themselves, but men did not catch up. Hence, the changing role of women is still social issue. The message is as follows: new is different, it does not mean worse, and hope for the future is fundamental.

The sixth theme is democracy. The authors explain the importance of including the structure of the population as a dimension to be considered for avoiding citizen detachment. The current democratic system, as an example, does not respect the composition of the population. Weighted votes based on age should be considered, since youths more than seniors will benefit and suffer the consequences of nowadays decisions. Gender is still an issue: despite quotas, parity has not been reached in terms of participation and sharing. Migrants contribute to the system but do not yet have the right to vote. Some people might be excluded once online mechanisms of voting are introduced. A final issue covered in this chapter is the necessity of a stronger investment in citizens’ ability to understand data, a pre-requisite to define our own future.

The final chapter is not a real conclusion but presents the last theme: sustainable development. It explains population dynamics, from the demographic transition due more to a reduction in mortality than to an increase in fertility, to the differences between poor and rich societies, where one of the solutions proposed to reduce fertility is to spread the Western concept of quality of children instead of quantity.

The lack of a proper conclusion leads the reader to personal considerations. The book is very ambitious: it covers several important topics related to the six themes. Every chapter is coherent with the aims of the book: highlighting the need to consider diverse population groups (the authors remind us that one person might belong to various groups), the importance of guaranteeing mechanisms for trusting, and finally the relevant role of individual, group-specific, and total wellbeing for a future outlook. Some chapters offer practical solutions; most of them explain and discuss the main keys that should be considered by politicians, economists, sociologists, and policymakers. This book helps demography to be salient in the definition of the public agenda.

